# Supplementary material for: Zebrafish Posterior Lateral Line primordium migration requires interactions between a superficial sheath of motile cells and the skin
Source: eLife. 2020 Nov 25;9:e58251. doi: 10.7554/eLife.58251 (PMC7688310; doi:10.7554/eLife.58251)
Supplement: Supplementary file 1. [file elife-58251-supp1.docx]

| Experiment | Observations | Embryos | Movies |
| --- | --- | --- | --- |
| DMSO superficial (Fig 3G) | 562 | 5 | 5 |
| SU5402 superficial (Fig 3I) | 303 | 5 | 5 |
| DMSO basal (Fig 3K) | 442 | 5 | 5 |
| SU5402 basal (Fig 3M) | 783 | 6 | 6 |
|  |  |  |  |
| Superficial intact (Fig 3B, 6B) | 440 | 5 | 5 |
| Superficial skinned (Fig 6D) | 181 | 6 | 6 |
| Superficial after healing (Fig 6F) | 529 | 5 | 5 |
|  |  |  |  |
| Basal intact (Fig 6I) | 646 | 10 | 10 |
| Basal skinned (Fig 6K) | 497 | 11 | 15 |
| Basal after healing (Fig 6M) | 761 | 6 | 8 |
|  |  |  |  |
| Matrigel superficial (Fig 7B) | 533 | 10 | 12 |
| Matrigel basal (Fig 7D) | 398 | 7 | 12 |
|  |  |  |  |
| Total | 6075 | 81 | 94 |
