# Supplementary material for: Zebrafish Posterior Lateral Line primordium migration requires interactions between a superficial sheath of motile cells and the skin
Source: eLife. 2020 Nov 25;9:e58251. doi: 10.7554/eLife.58251 (PMC7688310; doi:10.7554/eLife.58251)
Supplement: Source data 1. [file elife-58251-data1.zip › dallenogare2019-master/SAS_report_Fig7.pdf]

The SAS System

The CONTENTS Procedure

**Data Set Name**	DDN.DDN	**Observations**	231
**Member Type**	DATA	**Variables**	5
**Engine**	V9	**Indexes**	0
**Created**	09/26/2020 11:51:59	**Observation Length**	40
**Last Modified**	09/26/2020 11:51:59	**Deleted Observations**	0
**Protection**		**Compressed**	NO
**Data Set Type**		**Sorted**	NO
**Label**			
**Data Representation**	WINDOWS_64		
**Encoding**	wlatin1 Western (Windows)		

Engine/Host Dependent Information	
**Data Set Page Size**	65536
**Number of Data Set Pages**	1
**First Data Page**	1
**Max Obs per Page**	1632
**Obs in First Data Page**	231
**Number of Data Set Repairs**	0
**ExtendObsCounter**	YES
**Filename**	C:\ddn\ddn.sas7bdat
**Release Created**	9.0401M3
**Host Created**	X64_8PRO

Alphabetic List of Variables and Attributes

#	Variable	Type	Len	Format	Informat	Label
**4**	embryo	Num	8	BEST.		embryo
**2**	medium	Num	8	BEST.		medium
**5**	sample_ID	Num	8	BEST.		sample ID
**1**	skin	Char	6	$6.	$6.	skin
**3**	speed	Num	8	BEST.		speed

The SAS System

The Mixed Procedure

Model Information	
Data Set	DDN.DDN
Dependent Variable	speed
Covariance Structure	Variance Components
Subject Effect	embryo
Estimation Method	REML
Residual Variance Method	Profile
Fixed Effects SE Method	Model-Based
Degrees of Freedom Method	Containment

Class Level Information		
Class	Levels	Values
embryo	10	1 2 3 4 5 13 14 15 16 17
medium	2	1 2

Dimensions	
Covariance Parameters	2
Columns in X	3
Columns in Z per Subject	1
Subjects	10
Max Obs per Subject	11

Number of Observations	
Number of Observations Read	101
Number of Observations Used	101
Number of Observations Not Used	0

Iteration History			
Iteration	Evaluations	-2 Res Log Like	Criterion
0	1	-15.98297660	
1	2	-125.06929510	0.00000062
2	1	-125.06939086	0.00000000

Convergence criteria met.

Covariance Parameter Estimates					
Cov Parm	Subject	Estimate	Standard Error	Z Value	Pr > Z
Intercept	embryo	0.04287	0.02200	1.95	0.0257
Residual		0.01137	0.001686	6.75	<.0001

Fit Statistics	
-2 Res Log Likelihood	-125.1
AIC (Smaller is Better)	-121.1
AICC (Smaller is Better)	-120.9
BIC (Smaller is Better)	-120.5

Solution for Fixed Effects						
Effect	medium	Estimate	Standard Error	DF	t Value	Pr > |t|
Intercept		0.8071	0.09380	8	8.60	<.0001
medium	1	0.1124	0.1327	91	0.85	0.3991
medium	2	0	.	.	.	.

Solution for Random Effects						
Effect	embryo	Estimate	Std Err Pred	DF	t Value	Pr > |t|
Intercept	1	0.2302	0.09727	91	2.37	0.0200
Intercept	2	0.2192	0.09727	91	2.25	0.0266
Intercept	3	0.06883	0.09727	91	0.71	0.4810
Intercept	4	-0.2732	0.09727	91	-2.81	0.0061
Intercept	5	-0.2451	0.09727	91	-2.52	0.0135
Intercept	13	0.1294	0.09725	91	1.33	0.1866
Intercept	14	-0.03259	0.09725	91	-0.34	0.7383
Intercept	15	-0.2359	0.09725	91	-2.43	0.0173
Intercept	16	0.1423	0.09725	91	1.46	0.1468
Intercept	17	-0.00324	0.09694	91	-0.03	0.9734

Type 3 Tests of Fixed Effects				
Effect	Num DF	Den DF	F Value	Pr > F
medium	1	91	0.72	0.3991

The SAS System

The Mixed Procedure

Model Information	
Data Set	DDN.DDN
Dependent Variable	speed
Covariance Structure	Variance Components
Subject Effect	embryo
Estimation Method	REML
Residual Variance Method	Profile
Fixed Effects SE Method	Model-Based
Degrees of Freedom Method	Containment

Class Level Information		
Class	Levels	Values
embryo	10	6 7 8 18 19 20 21 22 23 24
medium	2	1 2

Dimensions	
Covariance Parameters	2
Columns in X	3
Columns in Z per Subject	1
Subjects	10
Max Obs per Subject	10

Number of Observations	
Number of Observations Read	100
Number of Observations Used	100
Number of Observations Not Used	0

Iteration History			
Iteration	Evaluations	-2 Res Log Like	Criterion
0	1	-82.78913139	
1	1	-114.21283752	0.00000000

Convergence criteria met.

Covariance Parameter Estimates					
**Cov Parm**	**Subject**	**Estimate**	**Standard Error**	**Z Value**	**Pr > Z**
**Intercept**	embryo	0.01120	0.006312	1.78	0.0379
**Residual**		0.01412	0.002105	6.71	<.0001

Fit Statistics	
**-2 Res Log Likelihood**	-114.2
**AIC (Smaller is Better)**	-110.2
**AICC (Smaller is Better)**	-110.1
**BIC (Smaller is Better)**	-109.6

Solution for Fixed Effects						
**Effect**	**medium**	**Estimate**	**Standard Error**	**DF**	**t Value**	**Pr > |t|**
**Intercept**		0.1998	0.04245	8	4.71	0.0015
**medium**	1	-0.1430	0.07751	90	-1.84	0.0683
**medium**	2	0	.	.	.	.

Solution for Random Effects						
**Effect**	**embryo**	**Estimate**	**Std Err Pred**	**DF**	**t Value**	**Pr > |t|**
**Intercept**	6	0.02066	0.06761	90	0.31	0.7607
**Intercept**	7	-0.08304	0.06761	90	-1.23	0.2225
**Intercept**	8	0.06239	0.06761	90	0.92	0.3586
**Intercept**	18	0.05540	0.05173	90	1.07	0.2870
**Intercept**	19	0.07362	0.05173	90	1.42	0.1581
**Intercept**	20	0.05637	0.05173	90	1.09	0.2787
**Intercept**	21	-0.08331	0.05173	90	-1.61	0.1108
**Intercept**	22	-0.2000	0.05173	90	-3.87	0.0002
**Intercept**	23	-0.00097	0.05173	90	-0.02	0.9851
**Intercept**	24	0.09889	0.05173	90	1.91	0.0591

Type 3 Tests of Fixed Effects				
**Effect**	**Num DF**	**Den DF**	**F Value**	**Pr > F**
**medium**	1	90	3.40	0.0683

The SAS System

The Mixed Procedure

Model Information	
Data Set	DDN.DDN
Dependent Variable	speed
Covariance Structure	Variance Components
Subject Effect	embryo
Estimation Method	REML
Residual Variance Method	Profile
Fixed Effects SE Method	Model-Based
Degrees of Freedom Method	Containment

Class Level Information		
Class	Levels	Values
embryo	11	1 2 3 4 5 6 7 8 9 11 12
skin	3	healed intact remove

Dimensions	
Covariance Parameters	2
Columns in X	4
Columns in Z per Subject	1
Subjects	11
Max Obs per Subject	10

Number of Observations	
Number of Observations Read	110
Number of Observations Used	110
Number of Observations Not Used	0

Iteration History			
Iteration	Evaluations	-2 Res Log Like	Criterion
0	1	-28.04968889	
1	1	-110.54986815	0.00000000

Convergence criteria met.

Covariance Parameter Estimates					
Cov Parm	Subject	Estimate	Standard Error	Z Value	Pr > Z
Intercept	embryo	0.03465	0.01807	1.92	0.0276
Residual		0.01485	0.002111	7.04	<.0001

Fit Statistics	
-2 Res Log Likelihood	-110.5
AIC (Smaller is Better)	-106.5
AICC (Smaller is Better)	-106.4
BIC (Smaller is Better)	-105.8

Solution for Fixed Effects						
Effect	skin	Estimate	Standard Error	DF	t Value	Pr > |t|
Intercept		0.05681	0.1098	8	0.52	0.6187
skin	healed	0.7649	0.1552	99	4.93	<.0001
skin	intact	0.8627	0.1388	99	6.21	<.0001
skin	remove	0	.	.	.	.

Solution for Random Effects						
Effect	embryo	Estimate	Std Err Pred	DF	t Value	Pr > |t|
Intercept	1	0.2266	0.08983	99	2.52	0.0132
Intercept	2	0.2158	0.08983	99	2.40	0.0182
Intercept	3	0.06775	0.08983	99	0.75	0.4525
Intercept	4	-0.2689	0.08983	99	-2.99	0.0035
Intercept	5	-0.2412	0.08983	99	-2.69	0.0085
Intercept	6	0.02230	0.1118	99	0.20	0.8423
Intercept	7	-0.08967	0.1118	99	-0.80	0.4245
Intercept	8	0.06736	0.1118	99	0.60	0.5482
Intercept	9	-0.1142	0.1118	99	-1.02	0.3097
Intercept	11	0.06631	0.1118	99	0.59	0.5544
Intercept	12	0.04785	0.1118	99	0.43	0.6696

Type 3 Tests of Fixed Effects				
Effect	Num DF	Den DF	F Value	Pr > F
skin	2	99	20.85	<.0001

The SAS System

The Mixed Procedure

Model Information	
Data Set	DDN.DDN
Dependent Variable	speed
Covariance Structure	Variance Components
Subject Effect	embryo
Estimation Method	REML
Residual Variance Method	Profile
Fixed Effects SE Method	Model-Based
Degrees of Freedom Method	Containment

Class Level Information		
Class	Levels	Values
embryo	13	1 2 3 4 5 9 11 12 13 14 15 16 17
skin	2	healed intact

Dimensions	
Covariance Parameters	2
Columns in X	3
Columns in Z per Subject	1
Subjects	13
Max Obs per Subject	11

Number of Observations	
Number of Observations Read	131
Number of Observations Used	131
Number of Observations Not Used	0

Iteration History			
Iteration	Evaluations	-2 Res Log Like	Criterion
0	1	-37.89334091	
1	2	-174.76819460	0.00000019
2	1	-174.76823358	0.00000000

Convergence criteria met.

Covariance Parameter Estimates					
Cov Parm	Subject	Estimate	Standard Error	Z Value	Pr > Z
Intercept	embryo	0.03578	0.01570	2.28	0.0113
Residual		0.01047	0.001363	7.68	<.0001

Fit Statistics	
-2 Res Log Likelihood	-174.8
AIC (Smaller is Better)	-170.8
AICC (Smaller is Better)	-170.7
BIC (Smaller is Better)	-169.6

Solution for Fixed Effects						
Effect	skin	Estimate	Standard Error	DF	t Value	Pr > |t|
Intercept		0.8633	0.06068	11	14.23	<.0001
skin	healed	-0.04159	0.1263	118	-0.33	0.7426
skin	intact	0	.	.	.	.

Solution for Random Effects						
Effect	embryo	Estimate	Std Err Pred	DF	t Value	Pr > |t|
Intercept	1	0.2842	0.06703	118	4.24	<.0001
Intercept	2	0.2732	0.06703	118	4.08	<.0001
Intercept	3	0.1233	0.06703	118	1.84	0.0684
Intercept	4	-0.2178	0.06703	118	-3.25	0.0015
Intercept	5	-0.1898	0.06703	118	-2.83	0.0054
Intercept	9	-0.1157	0.1123	118	-1.03	0.3050
Intercept	11	0.06719	0.1123	118	0.60	0.5507
Intercept	12	0.04848	0.1123	118	0.43	0.6667
Intercept	13	0.07447	0.06703	118	1.11	0.2688
Intercept	14	-0.08709	0.06703	118	-1.30	0.1964
Intercept	15	-0.2899	0.06703	118	-4.32	<.0001
Intercept	16	0.08735	0.06703	118	1.30	0.1951
Intercept	17	-0.05796	0.06649	118	-0.87	0.3851

Type 3 Tests of Fixed Effects				
Effect	Num DF	Den DF	F Value	Pr > F
skin	1	118	0.11	0.7426
