# Supplementary material for: Zebrafish Posterior Lateral Line primordium migration requires interactions between a superficial sheath of motile cells and the skin
Source: eLife. 2020 Nov 25;9:e58251. doi: 10.7554/eLife.58251 (PMC7688310; doi:10.7554/eLife.58251)
Supplement: Transparent reporting form [file elife-58251-transrepform.docx]

***eLife’s* transparent reporting form**

We encourage authors to provide detailed information *within their submission* to facilitate the interpretation and replication of experiments. Authors can upload supporting documentation to indicate the use of appropriate reporting guidelines for health-related research (see [EQUATOR Network](http://www.equator-network.org/%20)), life science research (see the [BioSharing Information Resource](https://biosharing.org/)), or the [ARRIVE guidelines](http://www.plosbiology.org/article/info:doi/10.1371/journal.pbio.1000412) for reporting work involving animal research. Where applicable, authors should refer to any relevant reporting standards documents in this form.

If you have any questions, please consult our Journal Policies and/or contact us: [editorial@elifesciences.org](mailto:editorial@elifesciences.org).

**Sample-size estimation**

- You should state whether an appropriate sample size was computed when the study was being designed
- You should state the statistical method of sample size computation and any required assumptions
- If no explicit power analysis was used, you should describe how you decided what sample (replicate) size (number) to use

Please outline where this information can be found within the submission (e.g., sections or figure legends), or explain why this information doesn’t apply to your submission:

No power calculations were performed to determine sample size. We targeted experiments to use a sample size of at least 5 individual embryos in each condition, based on our expectation of the large effect size for the manipulations described. For one experiment with a large effect (data in Figure 3G, Figure 7E), we performed the experiment on > 10 embryos, but used only 3 of these embryos for quantitative analysis (using all three embryos of the embryos which we generated and imaged for the explicit purpose of quantification).

**Replicates**

- You should report how often each experiment was performed
- You should include a definition of biological versus technical replication
- The data obtained should be provided and sufficient information should be provided to indicate the number of independent biological and/or technical replicates
- If you encountered any outliers, you should describe how these were handled
- Criteria for exclusion/inclusion of data should be clearly stated
- High-throughput sequence data should be uploaded before submission, with a private link for reviewers provided (these are available from both GEO and ArrayExpress)

Please outline where this information can be found within the submission (e.g., sections or figure legends), or explain why this information doesn’t apply to your submission:

All experiments were performed on a minimum of n=5 embryos, except for the data presented in Figure 3G/7H, as described above (n=3)). All outliers were included in the analysis. All the quantified data was included in statistical analysis; no quantified data or outliers were excluded.

**Statistical reporting**

- Statistical analysis methods should be described and justified
- Raw data should be presented in figures whenever informative to do so (typically when N per group is less than 10)
- For each experiment, you should identify the statistical tests used, exact values of N, definitions of center, methods of multiple test correction, and dispersion and precision measures (e.g., mean, median, SD, SEM, confidence intervals; and, for the major substantive results, a measure of effect size (e.g., Pearson's r, Cohen's d)
- Report exact p-values wherever possible alongside the summary statistics and 95% confidence intervals. These should be reported for all key questions and not only when the p-value is less than 0.05.

Please outline where this information can be found within the submission (e.g., sections or figure legends), or explain why this information doesn’t apply to your submission:

Statistical test and n samples used can be found in the appropriate figure legends. In all boxplots, all individual observations are included as a swarmplot. For protrusion directionality measurements, the number of embryos and observations used can be found in supplementary file 1.

(For large datasets, or papers with a very large number of statistical tests, you may upload a single table file with tests, Ns, etc., with reference to sections in the manuscript.)

**Group allocation**

- Indicate how samples were allocated into experimental groups (in the case of clinical studies, please specify allocation to treatment method); if randomization was used, please also state if restricted randomization was applied
- Indicate if masking was used during group allocation, data collection and/or data analysis

Please outline where this information can be found within the submission (e.g., sections or figure legends), or explain why this information doesn’t apply to your submission:

All embryos were randomly assigned to experimental or control groups. For each experiment, we used restricted randomization to keep the control and experimental groups approximately the same size.

**Additional data files (“source data”)**

- We encourage you to upload relevant additional data files, such as numerical data that are represented as a graph in a figure, or as a summary table
- Where provided, these should be in the most useful format, and they can be uploaded as “Source data” files linked to a main figure or table
- Include model definition files including the full list of parameters used
- Include code used for data analysis (e.g., R, MatLab)
- Avoid stating that data files are “available upon request”

Please indicate the figures or tables for which source data files have been provided:

We have provided all raw quantified data and jupyter notebooks used for generating figures and statistical analysis to github as documented in the materials and methods, with the exception of figure 7E. The raw data for this is available on github, but this analysis was done in SAS. We have provided the complete SAS report on github. We have also provided a zip archive containing all these files as a supplementary file.
